# Developing a Longitudinal Point-of-Care Ultrasound (POCUS) Curriculum for Internal Medicine Residents

**DOI:** 10.7759/cureus.86598

**Published:** 2025-06-23

**Authors:** Jenny Y Chen, Megan Trieu, Adam Custer, Patrick Holman, Rachel Sarnoff, Lindsey Ward, Beda Cha, Daniel Kahn

**Affiliations:** 1 Internal Medicine, University of California Los Angeles, Los Angeles, USA; 2 Pulmonary and Critical Care, University of California San Diego, San Diego, USA; 3 Pulmonary and Critical Care, University of California Los Angeles, Los Angeles, USA; 4 Cardiology, University of California Los Angeles, Los Angeles, USA

**Keywords:** medical education, medical residency, pocus (point of care ultrasound), residency, ultrasound, ultrasound education

## Abstract

Introduction

Point-of-care ultrasound (POCUS) is increasingly recognized as a valuable tool, enhancing diagnostic accuracy and reducing complications. Despite these benefits, barriers such as limited faculty training, equipment access, and time constraints hinder POCUS integration into internal medicine programs. We evaluated the feasibility of a multimodal, longitudinal ultrasound curriculum designed for internal medicine residents.

Methods

The POCUS Training Program (PTP) was implemented in an internal medicine residency program at a quaternary care center. This longitudinal curriculum comprised self-directed online modules, faculty-led didactic and scanning sessions, and the compilation of a portfolio. Program effectiveness was assessed through pre- and post-program surveys with five-point Likert scales measuring confidence with image acquisition and interpretation.

Results

Among the 50 initial program enrollees for the 2021-2022 academic year, only nine completed the curriculum, with two meeting full requirements including completing the image portfolio. Post-program surveys responses (derived from 11 respondents among 96 total program participants at the end of 2023, 11% response rate) indicated significant improvements in confidence with the cardiac and volume assessments. In-person didactics and independent scanning were rated as the most effective learning modalities. Time constraints, inconsistent equipment access, and POCUS experience variability among supervising senior residents and attendings were the most commonly cited barriers. Suggestions for improvement included more supervised image reviews, patient scanning sessions, and streamlined image-saving workflows.

Discussion

We demonstrated that implementing a longitudinal POCUS curriculum can be challenging with low completion rates for the full program, but such a curriculum has the potential to improve confidence with POCUS exams. Addressing barriers, including equipment accessibility and faculty training, is paramount. Integrating POCUS into core residency curricula and increasing faculty development may also improve efficacy. This pilot program serves as a scaffold for future iterations.

## Introduction

In recent years, point-of-care ultrasound (POCUS) has become more widely adopted as a diagnostic and procedural tool as there is growing evidence that POCUS improves diagnostic accuracy, decreases time to diagnosis, reduces procedural complications, improves patient satisfaction and shared diagnostic understanding, and reduces healthcare costs [1,2]. Despite these benefits, significant barriers remain for the integration of POCUS in internal medicine practice. While some medical schools have integrated ultrasound into their curriculum, residents have variable hands-on experience with ultrasound prior to intern year. A few internal medicine residency programs have published results of their program-wide implementation of POCUS education, including a large military residency which incorporated POCUS training into their residents’ scheduled academic time after a pilot program showed success with knowledge retention and improved confidence with ultrasound-guided procedures [3]. A university-based residency program also developed a separate POCUS track for a subset of its residents and reported high satisfaction levels and an increase in participants’ use and comfort with POCUS exams [4]. While it is evident that internal medicine residents benefit from increased POCUS training and practice opportunities, some limitations to ultrasound curriculum development and implementation include lack of trained faculty, insufficient equipment funding, and time constraints with patient care and other core educational requirements [5].

We designed a multimodal, longitudinal ultrasound curriculum to help internal medicine residents advance their POCUS skills. Our goal was to evaluate the feasibility and utility of implementing this elective program and provide a potential framework for other internal medicine residency programs to develop and integrate similar POCUS curricula.

## Materials and methods

POCUS Training Program (PTP) was implemented in an internal medicine residency program at an academic tertiary and quaternary care center during the 2021-2022 academic year. A study to evaluate the program was reviewed by the University of California, Los Angeles Institutional Review Board (IRB #21-001088). Inclusion criteria for this voluntary training program included being a current internal medicine or medicine-pediatric resident at any stage of residency training. There were no exclusion criteria.

The educational objectives of the program were the following:

1. Demonstrate understanding of the fundamentals of ultrasound operation and image optimization (“knobology”). 2. Identify appropriate indications for performing the following examinations: cardiac, volume status, pulmonary, deep vein thrombosis, abdominal, and renal. 3. Demonstrate proper technique in performing the above examinations and obtain clear and accurate images to allow for image interpretation. 4. Interpret images in the clinical context to recognize normal anatomy and abnormal pathology.

This longitudinal curriculum employed a blended learning approach that comprised self-directed online modules, internal medicine faculty-led didactic and scanning sessions, and the compilation of an ultrasound portfolio. Residents were instructed to review six self-paced, approximately one-hour-long online modules from the American College of Physicians prior to faculty-led sessions [6]. The modules were intended to allow residents to gain basic exposure to a specific ultrasound topic prior to a more detailed exploration of the topic with trained faculty. The two-hour-long faculty-led sessions occurred monthly and were held in the evenings after work hours to minimize scheduling conflicts with clinical duties. These sessions consisted of a lecture on a systems-based ultrasound topic followed by a supervised scanning session on ultrasound simulators and peer models. The didactics covered knobology, cardiac, lung, volume status, deep vein thrombosis (DVT), urinary, abdominal, and procedural guidance POCUS exams and included additional review sessions at the end of the year. These sessions were repeated the following year with the goal for residents to attend most, if not all, of the sessions by the end of the two-year period. Additionally, residents were given flash drives at the start of the program and encouraged to save their images to a cloud-based repository to build a portfolio for later individualized review with a trained instructor. Portfolio requirements included 20 cardiac, five pulmonary, five volume status, five DVT, three renal, and five abdominal studies, comprising both normal and pathological findings. Participants were able to request a review of their portfolio at any time by the POCUS program faculty for feedback on the quality and accuracy of the scans. In order to receive a certificate of completion, residents must have 1) attended a minimum of six unique core didactics, 2) completed all six online modules, 3) completed the ultrasound portfolio with faculty review, and 4) successfully passed a final hands-on exam.

The program was assessed with an anonymous survey administered to participants at the end of the program’s second year to residents who were actively enrolled in the program. The survey included five-point Likert scale questions with the aim to assess confidence levels with performing basic POCUS exams as well as the frequency of POCUS use, and included open-response questions to garner feedback on the program (Appendix).

## Results

Program participants

Among the 50 initial program enrollees for the 2021-2022 academic year, there were 22 first-year residents (44%), 21 second-year residents (42%), and seven third-year residents (14%) (Table 1). The most commonly noted future career plans were hospitalist (14 residents, 28%), cardiology (12 residents, 24%), and primary care (seven residents, 14%) (Table 1). Among this initial cohort, by the time of graduation, seven residents completed the curriculum portion but not the portfolio (14% of initial program enrollees), and two residents completed the full program including the portfolio (4% of initial program enrollees) (Table 1). A post-program feedback survey was administered to a total of 96 active program participants who were still enrolled in the program at the end of 2023 and had not yet graduated from residency. There were 11 survey respondents out of 96 program participants (11% response rate), comprising six first-year residents (55% of respondents), two second-year residents (18% of respondents), and three third-year residents (27% of respondents) (Table 2).

**Table 1:** PTP Baseline Survey Results, N = 50 The data has been represented as N and %. PTP: POCUS Training Program; DVT: Deep vein thrombosis

	n	%
Year in Training		
PGY-1	22	44%
PGY-2	21	42%
PGY-3	7	14%
Future Career Plans		
Hospitalist	14	28%
Cardiology	12	24%
Primary Care	7	14%
Hematology/Oncology	4	8%
Pulmonary Critical Care	4	8%
Gastroenterology	3	6%
Rheumatology	1	2%
Geriatrics	1	2%
Palliative Care	1	2%
Undecided	3	6%
Most Important Exams		
Cardiac	50	100%
Volume	50	100%
Lung	48	96%
Procedural	46	92%
Abdominal	44	88%
DVT	31	62%
Renal	28	56%
Soft Tissue	25	50%
Aorta	22	44%
Gallbladder	22	44%
Ophthalmology	5	10%
Program Completion		
Curriculum only	7	14%
Curriculum with portfolio	2	4%

**Table 2:** PTP Post-Program Feedback Survey Results, N=11 The data has been represented as N and %. Year in Training refers to the current PGY year of the respondent. PTP: POCUS Training Program; DVT: Deep vein thrombosis

	n	%
Year in Training		
PGY-1	6	55%
PGY-2	2	18%
PGY-3	3	27%
Most Often Used Exams		
Cardiac	9	82%
Volume	8	73%
Procedures	5	45%
Lung	3	27%
DVT	1	9%
Frequency of POCUS Use		
Often (1-2 times/week)	3	27%
Sometimes (1-3 times/month)	6	55%
Rarely (<1 time/month)	2	18%

Needs assessment

When the initial program cohort was polled on which types of exams were most important for future internists to be comfortable with, participants noted the following top five exams in descending order of importance: cardiac, volume, lung, procedural guidance, and abdominal free fluid (Table 1). Residents noted that they most often used ultrasound for cardiac (nine of 11 residents, 82%) and volume (eight of 11 residents, 73%) assessments as well as for procedural guidance (five of 11 residents, 45%) (Table 2). Around half of the resident respondents (six of 11 residents, 55%) noted that they incorporate ultrasound into their practice one to three times per month with another quarter of the group (three of 11 residents, 27%) noting that they use ultrasound one to two times per week (Table 2).

Modes of learning

In-person didactics and independent scanning for the portfolio were seen as the most effective modes of learning with nine of 11 respondents to the post-program survey (82%) labeling the in-person didactics as effective or very effective, and all 11 respondents (100%) labeling independent scanning as effective or very effective.

Confidence levels with performing POCUS exams

In the post-program survey, residents reported being most confident with using ultrasound for volume assessment and procedural guidance with eight residents (73%) and nine residents (82%) out of 11 reporting being completely or fairly confident in these exams, respectively. Residents were least comfortable with the DVT, abdominal, and urinary exams with only one (9%) or two (18%) of the 11 residents reporting being completely or fairly confident in each exam. Chi-square analysis of Likert scale scores was conducted to compare pre- and post-program confidence levels with each exam. Lower confidence level responses (“not at all confident,” “slightly confident,” and “somewhat confident”) were pooled together and compared against higher confidence level responses (“fairly confident” and “completely confident”). A p-value of 0.05 or lower was considered statistically significant. There were statistically significant improvements in confidence with the cardiac and volume assessments after participation in the program (Table 3).

**Table 3:** Confidence with POCUS Exams Pre- and Post-Program The data has been represented as N and % pre- and post-program. Chi-square tests were used with a p<0.05 considered statistically significant. DVT: Deep vein thrombosis

Exam	Pre, n	Pre, %	Post, n	Post, %	Chi-square	p-value
Cardiac					8.89	0.031
Not at all confident	17	34%	0	0%		
Slightly confident	16	32%	5	45%		
Somewhat confident	9	18%	1	9%		
Fairly confident	5	10%	4	36%		
Completely confident	3	6%	1	9%		
Lung					2.45	0.484
Not at all confident	22	44%	3	27%		
Slightly confident	13	26%	2	18%		
Somewhat confident	6	12%	3	27%		
Fairly confident	8	16%	3	27%		
Completely confident	1	2%	0	0%		
Volume					12.77	0.005
Not at all confident	13	26%	0	0%		
Slightly confident	10	20%	1	9%		
Somewhat confident	13	26%	2	18%		
Fairly confident	13	26%	7	64%		
Completely confident	1	2%	1	9%		
DVT					4.29	0.232
Not at all confident	37	74%	4	36%		
Slightly confident	8	16%	4	36%		
Somewhat confident	4	8%	2	18%		
Fairly confident	1	2%	1	9%		
Completely confident	0	0%	0	0%		
Urinary					4.86	0.182
Not at all confident	35	70%	4	36%		
Slightly confident	7	14%	4	36%		
Somewhat confident	5	10%	1	9%		
Fairly confident	2	4%	2	18%		
Completely confident	1	2%	0	0%		
Abdominal					2.53	0.470
Not at all confident	26	52%	5	45%		
Slightly confident	6	12%	3	27%		
Somewhat confident	9	18%	2	18%		
Fairly confident	5	10%	1	9%		
Completely confident	4	8%	0	0%		

Barriers to POCUS use

The most commonly reported barrier to POCUS use during clinical practice was time constraints, which was noted by all 11 (100%) of the respondents. About a third of respondents (three of 11 residents, 27%) also noted a lack of consistent access to ultrasound machines as an additional challenge. Other cited barriers included apprehension with using the ultrasound machines (knobology) as well as perceived inexperience among senior residents and attendings with teaching and supervising POCUS on the wards with real patient cases. Most residents (nine of 11 residents, 82%) noted that they rarely or never save their ultrasound images.

Feedback and areas for improvement

Free text comments from survey respondents demonstrated overall satisfaction with the program, particularly with the didactic sessions. For areas for improvement, respondents noted interest in more supervised image review opportunities to improve their imaging techniques and more hands-on supervised practice with real patients to gain exposure to pathological findings and to supplement the group scanning sessions on ultrasound simulators and other program participants.

## Discussion

This pilot POCUS training program demonstrates both the potential and challenges of implementing ultrasound education in internal medicine residency programs. Overall, participants found both the faculty-led didactics and independent scanning for the portfolio to be effective modes of learning. The program’s blended learning approach led to high engagement among residents based on general verbalization during the program and overall positive sentiments among free text comments from the post-program survey, highlighting the demand for such training. The most attended didactic sessions occurred earlier in the program and correlated with the exams that participants noted were most important for internists. Accordingly, by the end of the program, participants described the greatest confidence in the cardiac and volume exams, with statistically significant improvement in confidence levels for both exams. Ultimately, however, the evaluation of the effectiveness of the components of PTP overall was likely limited by the low response rate in the post-program survey. Survey responses may have incurred some selection bias with survey participants potentially being those with greater engagement with the program overall as well as those with higher confidence with POCUS in general.

Several barriers impeded program completion and full utilization of ultrasound in clinical practice, with only nine out of 50 initial participants completing the curriculum (18%) and only two of those nine completing the full program including the image portfolio (22% of those who completed the curriculum, 4% completion rate for PTP overall). Post-program survey feedback revealed that the most common barriers to more widespread POCUS use were time constraints and lack of consistent access to ultrasound machines. These results mirrored the study of another university-based POCUS track for internal medicine residents, which noted time constraints (83% of their survey participants) and lack of available ultrasound equipment (83%) as the greatest barriers for more widespread POCUS use [4]. Teaching abbreviated but high-yield exams, especially those geared towards informing acute management decisions, could help address these perceived time constraints. Examples include emphasizing use of established POCUS applications such as the eFAST protocol for trauma and the BLUE protocol for assessment of respiratory failure and providing additional instruction on the focused cardiac ultrasound exam (FoCUS), which took residents an average of 3.8 minutes to complete in a study of resident cardiac exams in emergency departments [7-9]. Improving awareness of available equipment and optimizing accessibility while maintaining security is also crucial. Handheld ultrasound machines are rising in popularity and present another avenue for more widespread POCUS use with their portability [10].

Another major obstacle to POCUS use by trainees was the perceived variability in POCUS experience among supervising residents and attendings. The lag in ultrasound training for hospitalists has created an educational bottleneck, underscoring the need to dedicate resources for training hospitalist faculty using published curricula designed for experienced clinicians [11-13]. A parallel focus on recruiting and training dedicated internal medicine attendings in POCUS, in addition to training residents, was highlighted in the assessment of a POCUS training program at another large academic institution with similar hopes of expanding POCUS education and supervision until POCUS becomes a standard, integrated tool in clinical care [14].

Saving images to build a portfolio ultimately proved to be the most challenging component of PTP. This mirrors the clinical issue of "phantom scans," where ultrasounds are performed but not saved, which can negatively affect patient care [15]. Building an ultrasound portfolio offers the opportunity for spaced repetition of POCUS maneuvers and has been correlated with improved skills retention over time, as demonstrated in the outcome analysis of an ultrasound training program among hospitalists at a tertiary care medical center [16]. To address the challenges noted by our program participants and empower the completion of the portfolio, we plan to incorporate more training on machine knobology, streamline the image-saving workflow, and post instructions on how to save in resident work rooms and on ultrasound machines. As POCUS becomes further integrated into medical school curricula and even in the pre-clinical clerkship years, future residents who enroll in PTP may bring prior foundational experience with POCUS knobology and be able to provide further peer-to-peer instruction and support during PTP sessions and portfolio building [17].

To enhance the completion rate and effectiveness of the program, we propose several improvements in addition to those mentioned above. Scheduling more in-person sessions with hands-on scanning of patients would increase practice with image acquisition and also help learners to recognize pathology in real-time. Adding faculty-led group image review sessions can boost image interpretation and clinical integration skills. Finally, although evening sessions allowed participation outside clinical schedules, these placed additional demands on residents’ time amidst existing clinical work. Ideally, POCUS should be integrated into the core residency curriculum during already protected educational time during standard work hours. Future directions for research on the effectiveness of PTP include gathering and analyzing longitudinal skills data for program participants, beginning with a baseline assessment at the start of the program and including interval assessments, in addition to the final hands-on exam already included in the completion criteria for the program.

## Conclusions

In conclusion, while our POCUS training program showed promise in engaging a large proportion of residents, a low post-program survey response rate limits our ability to draw reliable conclusions on the effectiveness of the program, which would be best evaluated in the future with additional longitudinal skills assessments among participants. The low completion rate of the program also highlighted the challenges of implementing a longitudinal POCUS program and the paramount need to address various barriers to POCUS use, which included time constraints, insufficient access to ultrasound equipment, and lack of comfort with saving images. This pilot program serves as a scaffold for future iterations both at our institution as well as at others, with the ultimate goal of producing confident and competent POCUS practitioners who can effectively incorporate this valuable tool into patient care.

## Appendices

PTP Post-Program Feedback Survey

1. What is your current level of training?

PGY1 PGY2 PGY3 PGY4

2. Which PTP sessions did you attend (include both the current academic year and prior years)? Select all that apply.

Introduction & Knobology Cardiac Lung DVT Abdominal Renal Procedures

3. How often do you use POCUS in the clinical setting?

Never Rarely (< 1 time per month) Sometimes (1-3 times per month) Often (1-2 times per week) Always (3 or more times per week)

4. Which of the following POCUS exams do you perform most frequently? Select up to 3 exams.

Abdominal Aorta Cardiac Gallbladder DVT Lung Ophthalmologic Procedural guidance Renal Soft tissue/MSK Volume status Other

5. How often are you saving your ultrasound images (on Butterfly, USB drive, or in Epic)?

Never Rarely (~25% of the time) Sometimes (~50% of the time) Usually (~75% of the time) Almost always or always (~90-100% of the time)

6. Rate your confidence level in acquiring and interpreting ultrasound images for the cardiac exam.

Not confident at all Slightly confident Somewhat confident Fairly confident Completely confident

7. Rate your confidence level in acquiring and interpreting ultrasound images for the volume status exam.

Not confident at all Slightly confident Somewhat confident Fairly confident Completely confident

8. Rate your confidence level in acquiring and interpreting ultrasound images for the lung exam.

Not confident at all Slightly confident Somewhat confident Fairly confident Completely confident

9. Rate your confidence level in acquiring and interpreting ultrasound images for the DVT exam.

Not confident at all Slightly confident Somewhat confident Fairly confident Completely confident

10. Rate your confidence level in acquiring and interpreting ultrasound images for the abdominal exam.

Not confident at all Slightly confident Somewhat confident Fairly confident Completely confident

11. Rate your confidence level in acquiring and interpreting ultrasound images for the renal exam.

Not confident at all Slightly confident Somewhat confident Fairly confident Completely confident

12. Rate your confidence level in acquiring and interpreting ultrasound images for procedural guidance.

Not confident at all Slightly confident Somewhat confident Fairly confident Completely confident

13. Rate the effectiveness of the following teaching formats in PTP as very effective, effective, neutral, ineffective, very ineffective, or did not utilize this resource:

Web-based module In-person didactics Supervised scanning Independent scanning Image review with instructor

14. What are the barriers to using POCUS when you are on wards? Select all that apply.

Time constraints with other clinical duties Lack of access to a Butterfly or Sonosite ultrasound Lack of comfort or confidence with using the ultrasound machine (using the buttons/capturing images, etc.) I do not feel comfortable using POCUS exams to guide clinical management (i.e., would rather obtain formal radiology studies) My senior residents (if applicable) often do not feel comfortable performing/supervising/teaching POCUS My attendings often do not feel comfortable performing/supervising/teaching POCUS

15. Please share any additional feedback or suggestions.
